# Clinical Profile and Evaluation of Outcomes of Symptomatic Gallstone Disease in the Senior Citizen Population

**DOI:** 10.7759/cureus.28492

**Published:** 2022-08-28

**Authors:** Mahendra Lodha, Anupam S Chauhan, Ashok Puranik, Satya Prakash Meena, Mayank Badkur, Ramkaran Chaudhary, Indra Singh Chaudhary, Metlapalli V Sairam, Vinod Kumar, Rashi Lodha

**Affiliations:** 1 General Surgery, All India Institute of Medical Sciences, Jodhpur, Jodhpur, IND; 2 Surgery, All India Institute of Medical Sciences, Jodhpur, Jodhpur, IND; 3 Medicine, Dr. Sampurnand Medical College, Jodhpur, IND

**Keywords:** acute cholangitis, gallstone cholecystitis, biliary pancreatitis, cholecystectomy, elderly, geriatric, gallstones, cholelithiasis

## Abstract

Background

There is a heavy burden of gallstone disease on the world’s population. The incidence and severity of symptomatic cholelithiasis increase with age. There is often a delay in presentation, leading to complicated disease, diagnostic delay, and increased morbidity. There is a paucity of studies on the presentation and management of cholelithiasis in elderly persons from the western part of India. This study aimed to observe the spectrum of presentation and management of symptomatic cholelithiasis in senior citizens.

Objectives

The primary objective of this study was to describe the presentation, diagnosis and intraoperative findings of symptomatic gallstone disease (GSD) in patients aged over 60 years. The secondary objectives of this study were to find the association of GSD with age, sex, and comorbidities, including diabetes mellitus, hypertension, and thyroid disorders.

Methods

All patients above the age of 60 years presenting to the surgical outpatient and emergency departments from January 2020 to July 2021 with symptomatic GSD were included. Details of history, physical examination, blood investigations, and imaging of the abdomen (ultrasonography and Magnetic Resonance Cholangiopancreaticography, when indicated) were recorded. Patients were managed as per the advice of the treating consultant. Details of management and outcomes, including hospital stay, mortality, and morbidity, were noted. The descriptive data were organised into tables and percentages. The significance of various data and relationships between various variables was analysed using the Pearson chi-square test, Fischer exact test and scatter plots.

Results

A total of 76 patients were evaluated in this study, of which 73.7% were female. The mean age was 70.8 ± 1.7 years. The majority of patients (63.2%) were admitted through the outpatient department (OPD). The most common presenting complaint was abdominal pain (96.1%). Clinical jaundice was noted in 9.2%. Complicated Gall Stone Disease (GSD) was found more commonly in the female population (57.1%). Complicated GSD was more commonly found in patients with diabetes (p=0.075) and hypothyroidism (p=0.057). No association of age with intraoperative complications was noted (p = 0.446).

Conclusion

Senior citizens can present with both complicated and uncomplicated GSD. GSD, in the presence of hypothyroidism or diabetes mellitus, presents in a much more complicated form. Early surgical intervention in form of laparoscopic cholecystectomy can be beneficial to the patient if diagnosed with symptomatic gallstones. Patients of this age group need not be over investigated if a benign pathology is suspected.

## Introduction

Cholelithiasis is a widespread disease, with prevalence rates of around 20% in adults [1]. As the age increases the prevalence of gallstone disease (GSD) increases from 8% to 50% in patients older than 70 years of age [2]. Women over the age of 70 years have the highest prevalence [3]. Gallstone-related complications like cholecystitis, choledocholithiasis and biliary pancreatitis are known to increase as age increases [4]. Gallstone formation has a multifactorial etiology from age, gender, and race to obesity, rapid weight loss, drugs, pregnancy and triglyceridemia. Based on these factors - four factors are known that cause gallstone formation - supersaturation of cholesterol in bile, cholesterol precipitation and crystallization, impaired gallbladder functions like contraction, motility and impaired bile reabsorption in the bowel. Because of increased age, there is hypomotility of the gallbladder because of sclerotic changes in the wall of the gallbladder. An increase in gallbladder volume causes impaired gallbladder motility and stasis of bile which causes precipitation of stones [5].

The current standard of treatment for symptomatic GSD in elderly patients is laparoscopic cholecystectomy. Studies have shown that old age is not a risk factor for poor outcomes in patients undergoing cholecystectomy [6].

However, there is still reluctance in elderly, old patients and their relatives, and even among some anaesthetists and surgeons, to proceed with early laparoscopic cholecystectomy, which is defined as cholecystectomy done within index admission, and interval laparoscopic cholecystectomy, defined as cholecystectomy done after four to six weeks of symptoms [7,8]. There has not been any significant research in the Indian subcontinent on the senior citizen population in this regard.

## Materials and methods

Study setting

This hospital-based prospective observational study was conducted on patients who were more than the age of 60 years of age at the time of admission and presented with symptomatic GSD to the Department of General Surgery, All India Institute of Medical Sciences (AIIMS), Jodhpur.

Participants

All patients who underwent elective laparoscopic cholecystectomy for symptomatic gall stones in the Department of General Surgery, AIIMS Jodhpur were recruited for study based on inclusion and exclusion criteria as mentioned below.

Inclusion Criteria

Patients belonging to the senior citizen population (>60 years of age) and diagnosed to have symptomatic GSD with or without polyps, with any signs or symptoms suggestive of gallstone-induced pancreatitis, cholangitis and obstructive jaundice were included in the study.

Exclusion Criteria

Any patients with non-gallstone induced pancreatitis, cholangitis and obstructive jaundice or with a diagnosis of carcinoma gallbladder were excluded from the study.

Sample size calculation

All patients presenting during a time span of 1.5 years from January 1, 2020 to July 31, 2021 were included in the study.

Study procedure

A protocol was drawn up and initially submitted to and cleared by the Institutional Review Board (IRB). Cases were recruited based on inclusion and exclusion criteria. Informed written consent was obtained. All patients were appropriately examined, and blood investigations like haemoglobin, total leukocyte counts, platelet counts, liver function tests, Hb1Ac and thyroid function tests were taken at the time of presentation. Radiological studies like ultrasound abdomen and MRCP (Magnetic resonance cholangiopancreatography) were conducted as indicated. In patients who had associated CBD stones, endoscopic retrograde cholangiopancreatography (ERCP) and stone retrieval were done if indicated, followed by laparoscopic cholecystectomy. Intra-operative findings of the surgery were noted. Gallbladder and gallstones were sent for pathological analysis and their final histopathological analysis was noted. Patients were followed up for a period of two weeks after surgery. Investigations like haemoglobin, total leucocyte count, platelet count, liver function tests, and thyroid function tests were repeated on follow-up if required. All the data were appropriately analysed.

Statistical analysis

Data were entered and analysed using SPSS version 2828 (IBM Corp., Armonk, NY). The nominal data were described using frequency and percentages and compared using the chi-square test or Fischer exact test. The ordinal data were described using median and interquartile range (IQR) and compared using the Mann-Whitney U test. The continuous data were described using mean +/- SD and compared using an unpaired t-test. A P-value of <0.05 will be considered statistically significant.

Ethical clearance

Ethical clearance was obtained from the Institutional Ethics Committee, AIIMS Jodhpur. The certificate reference number is AIIMS/IEC/2019-20/1003.

## Results

In the period of 1.5 years from January 1, 2020 to July 31, 2021, a total of 76 patients were recruited for the study who all matched the inclusion criteria of this study.

Out of 76 patients, 26.3% were male and 73.7% were females. The study population suffered from co-morbidities like diabetes mellitus (19.7%), hypertension (30.3%) or thyroid disorder (9.2%). Around 15.8% had a history of smoking and 30.3% had a history of alcohol consumption. A total of 63.2% of the study population were admitted to the hospital through the out-patient department (OPD) while 36.8% were admitted through the emergency department (Table 1).

**Table 1:** Demographic details of the study population ASA (American Society of Anaesthesiologists physical status classification)

Demographic variables		n (%)
Gender	Male	20 (26.3)
	Female	56 (73.7)
Age (years)	70.9 ± 2.0 (Females); 70.4 ± 3.1 (Males)	
Marital status	Married	74 (97.4)
	Unmarried	2 (2.6)
Educational status	Illiterate	66 (86.8)
	Literate	10 (13.2)
Occupation	Employed	7 (9.2)
	Unemployed	69 (90.8)
Comorbidities	Diabetes Mellitus	15 (19.7)
	Hypertension	23 (30.3)
	Thyroid Disorder	7 (9.2)
	Smoker	12 (15.8)
	Alcoholic	23 (30.3)
Admission type	OPD	48 (63.2)
	Emergency	28 (36.8)
ASA	1	20 (35.1)
	2	15 (26.3)
	3	21 (36.8)
	4	1 (1.8)
	5	0 (0)

The most common presenting complaints were abdominal pain (96.1%) with psia (60.5%) and vomiting (55.3%). Some of the patients also complained of loss of appetite (47.4%) and back pain (32.9%). The least common symptoms were fever (23.7%) and yellowish discoloration of the skin (9.2%). On clinical evaluation, 41.6% of patients had a positive Murphy’s Sign and 22.1% of the patients had abdominal distention. AbdomThe abdominal was only found in 5.2% of the patients.

**Figure 1:**
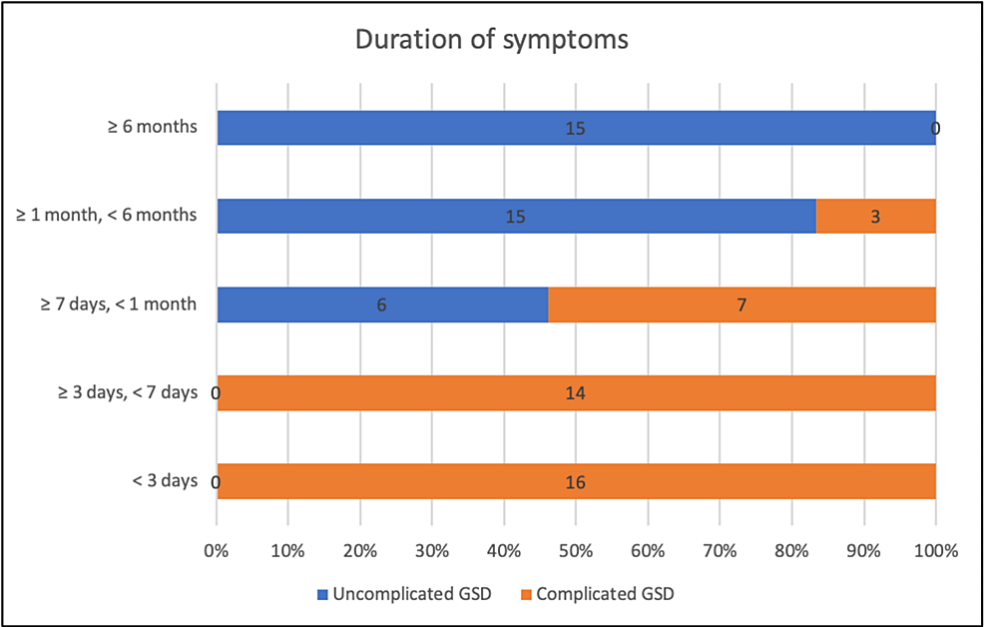
Duration of symptoms GSD - Gallstone Disease

Uncomplicated GSD like biliary colic presented with a trend towards a longer duration of symptomatic history, whereas complicated GSD (acute calculus cholecystitis, choledocholithiasis, biliary pancreatitis, cholangitis) presented to the hospital with a shorter duration of symptomatic history (Figure 1).

Out of 76, 74 patients underwent ultrasonographic evaluation of the abdomen where the following gallbladder findings were noted. A total of 47.3% of the 74 patients had thickened gallbladder walls. Around 37.8% of the patients had the presence of pericholecystic fluid. A total of 81.1% of the patients had multiple gallstones in the gallbladder while only 17.6% had a single stone. The average size of the single stone was 10.65 mm whereas the average size of the multiple stones was 6.85 mm. Around 20.3% of the 74 patients had dilated CBD (≥8 mm), and out of these only four patients had CBD stones on ultrasound. The average size of the CBD stones was 7.84 mm (Table 2). Out of 75 ultrasonographic evaluations, one study was not able to find the presence of gallstones. More than 80% of patients had multiple gallstones and had presented more commonly with biliary colic, acute calculus cholecystitis and choledocholithiasis.

**Table 2:** Ultrasonographic findings of the gallbladder, and their distribution among the patient population CBD - Common Bile Duct, mm - millimetres

Ultrasonographic findings of Gallbladder		No. of patients	Percentage
Gall bladder wall thickness (mm)	≤3	39	52.7
	>3	35	47.3
Pericholecystic fluid	Present	28	37.8
	Absent	46	62.2
No. of stone in Gallbladder	No stone	1	1.4
	Single	13	17.6
	Multiple	60	81.1
Stone size (mean)	Single (mm)	10.6	-
	Multiple (mm)	6.8	-
CBD diameter (mm)	<8	59	79.7
	≥8	15	20.3
CBD stone	Single	3	4.1
	Multiple	1	1.4
CBD stone size mean (mm)		7.84	-

Various blood investigations were done on admission. A total of 56.6% of patients had anaemia, and 31.6% had leucocytosis. On evaluating the liver function tests - 34.2% had deranged serum glutamate-pyruvate transaminase (SGPT) with only 25% with deranged serum glutamic oxaloacetic transaminase (SGOT). Around 10.5% of the study population had hyperbilirubinemia, and 27.6% had increased alkaline phosphatase (ALP). Lipase and amylase were increased in 22.4% and 13.2% of the study population, respectively. A total of 60.5% of the patients were found to have diabetes. More than 50% of the population studied had hypothyroidism.

Benign GSDs were classified into a spectrum of 5 clinical presentations - acute calculous cholecystitis, biliary colic, cholelithiasis with choledocholithiasis, biliary pancreatitis and cholangitis. The most common presentation was biliary colic (49.4%) followed by acute calculus cholecystitis (27.3%), while the least common was cholangitis (2.6%) and cholelithiasis with choledocholithiasis (6.5%). The incidence of biliary pancreatitis was higher (22.9%) in patients aged 60-69 years compared with those aged 70 years and above. Although the difference was not statistically significant, there was a trend towards significance (Table 3).

**Table 3:** Clinical presentation of gallstone disease and distribution in age groups (Pearson Chi-Square Test) GSD - Gall Stone Disease

Clinical presentation	Age (years)			Total	P-value
	60-69	70-79	≥80		
	n (%)	n (%)	n (%)	n (%)	
Biliary Colic	17 (48.6)	12 (42.9)	9 (69.2)	38 (50)	0.283
Acute Calculous Cholecystitis	7 (20)	11 (39.3)	3 (23.1)	21 (27.6)	0.217
Cholelithiasis with choledocholithiasis	2 (5.7)	3 (10.7)	0 (0)	5 (6.6)	0.419
Biliary Pancreatitis	8 (22.9)	1 (3.6)	1 (7.7)	10 (13.2)	0.064
Cholangitis	1 (2.9)	1 (3.6)	0 (0)	2 (2.6)	0.796
Uncomplicated GSD	16 (44.4)	11 (30.6)	9 (25.0)	36 (100.0)	0.195
Complicated GSD	19 (47.5)	17 (42.5)	4 (10.0)	40 (100.0)	

Uncomplicated GSD (biliary colic) was found to be more common in the male population as compared to the female population. Complicated GSD (acute calculus cholecystitis, cholelithiasis with choledocholithiasis, biliary pancreatitis, cholangitis) were found to be more common among the female population, though statistically not significant.

Out of 76 patients, 43.4% were found to be euthyroid, 52.6% were found to be suffering from hypothyroidism, and only 4% were found to have hyperthyroidism. Statistically, there was no significant difference found between the thyroid status of the patient and the clinical presentation but there was a significantly higher incidence of biliary pancreatitis associated with hypothyroidism. Patients who had diabetes were found to be associated with complicated GSD (60.9%), though it was found not to be statistically significant (Table 4).

**Table 4:** Correlation between comorbidities and gallstone clinical presentations Fisher Exact Test*; Pearson Chi-Square Test**

Co-morbidities		Clinical presentation		Total	P-value
		Uncomplicated GSD	Complicated GSD		
		n (%)	n (%)	n (%)	
Thyroid Disorders	Euthyroid	18 (54.5)	15 (45.5)	33 (100.0)	0.057*
	Hypothyroidism	15 (37.5)	25 (62.5)	40 (100.0)	
	Hyperthyroidism	3 (100.0)	0 (0.0)	3 (100.0)	
Hypertension	Hypertensive	9 (39.1)	14 (60.9)	23 (100.0)	0.343**
	Normotensive	27 (50.9)	26 (49.1)	53 (100.0)	
Diabetes	Diabetic	18 (39.1)	28 (60.9)	46 (100.0)	0.075**
	Not-diabetic	18 (60.0)	12 (40.0)	30 (100.0)	

A total of 15 patients underwent MRCP, of which 11 patients were found to have a dilated CBD (≥8 mm), and seven patients were found to have either CBD stone or sludge in the CBD. Out of these 11 patients, nine underwent ERCP stenting and stone retrieval, eight underwent laparoscopic cholecystectomy and one underwent exploratory laparotomy (Table 5).

**Table 5:** Findings of the biliary tree on MRCP MRCP - Magnetic resonance cholangiopancreatography, CBD - Common Bile Duct

MRCP Details		No. of patients	Percentage
Gallbladder stone number	Single	1	6.7
	Multiple	14	93.3
CBD diameter (mm)	<8	4	26.7
	≥8	11	73.3
CBD stone	No stone	8	53.3
	Single	2	13.3
	Multiple	1	6.7
	Sludge	4	26.7

Patients were managed via various modalities based on the condition of the patients. About 75% of the study population underwent some type of surgical intervention. A total of 11 patients were managed initially non-operatively and were planned for interval cholecystectomy, but due to the COVID-19 pandemic these patients were lost to follow-up. Due to uncontrolled diabetes, the surgery of one patient was deferred and the patient was ultimately lost to follow-up (Table 6).

**Table 6:** Different management plans for GSD ERCP - Endoscopic retrograde cholangiopancreatography

Management	n (%)
Non Operative Management	11 (14.5)
ERCP	6 (7.9)
Early Cholecystectomy	45 (59.2)
ERCP followed by Early Cholecystectomy	3 (3.9)
Interval Cholecystectomy	4 (5.3)
ERCP followed by Interval Cholecystectomy	4 (5.3)
Cholecystostomy	1 (1.3)
Exploratory Laparotomy	1 (1.3)
Deferred Surgery	1 (1.3)
Total	76 (100.0)

Out of 76 patients, 56 underwent laparoscopic cholecystectomy and one underwent open cholecystectomy, with or without ERCP. A total of 19 patients were managed non-operatively. Eleven had medical management only. Six had ERCP stenting. One had cholecystotomy done. One was planned for laparoscopic cholecystectomy, but their surgery was deferred due to COVID-19 quarantine protocol and was later lost to follow-up.

**Figure 2:**
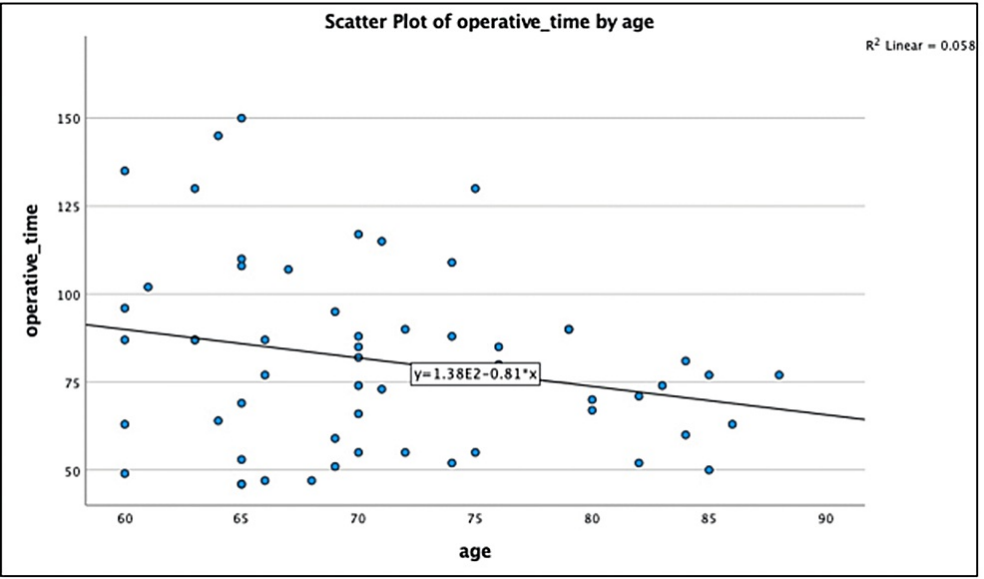
Operative time vs age (Scatter plot)

The patients were divided into three sub-groups based on age. There was no significant difference found between the groups in terms of the presence of adhesions, distension or contraction of the gallbladder, the occurrence of complications, conversion rate or use of drains in various age groups. There was also no significant difference in the mean operative time among the three groups, but there was a trend toward a shorter duration of surgery as the age increased. There was a statistically significant result noted in terms of the presence of adhesions, and the gross presentation of the gallbladder disease when the patients were divided into two groups based on their clinical presentation. The mean operative time, as well as the mean day of drain removal, was also significantly lesser in the patients suffering from uncomplicated GSD. When the operative details were evaluated based on the different management plans, adhesions were found to be more common in patients who had undergone ERCP and those who underwent interval cholecystectomy. Similarly, the gallbladder was found to be contracted in the same group of patients. Because of this, there was a significant increase in the operative time and day of removal of the drain in the postoperative period (Table 7, Figure 2).

**Table 7:** Operative details vs age groups, clinical presentation and different management approaches (Pearson Chi-Square Test) GSD - Gallstone Disease, EC - Early cholecystectomy, IC - Interval Cholecystectomy, ERCP - Endoscopic retrograde cholangiopancreatography

Operative Details		Age (yrs.)					Clinical presentation				Management					
		60-69	70-79	≥80	Total	P-value	Uncomplicated GSD	Complicated GSD	Total	P-value	EC	ERCP + EC	IC	ERCP + IC	EL	P-value
Adhesions	Present	11 (42.3)	11 (55.0)	4 (36.4)	26 (45.6)	0.547	7 (21.2)	19 (79.2)	26 (45.6)	<0.001	15 (33.3)	2 (66.7)	4 (100.0)	4 (100.0)	1 (100.0)	0.007
	Absent	15 (48.4)	9 (45.0)	7 (63.6)	31 (54.4)		26 (78.8)	5 (20.8)	31 (54.4)		30 (66.7)	1 (33.3)	0 (0)	0 (0)	0 (0)	
Gallbladder status	Distended	16 (64.0)	12 (60.0)	7 (63.7)	35 (61.4)	0.959	28 (84.8)	7 (29.2)	35 (61.4)	<0.001	34 (75.6)	0 (0)	1 (25)	0 (0)	0 (0)	<0.001
	Contracted	9 (36.0)	8 (40.0)	4 (36.3)	21 (36.8)		5 (15.2)	17 (70.8)	22 (38.6)		11 (24.4)	3 (100)	3 (75)	4 (100)	1 (100.0)	
Complication	Occurred	2 (7.7)	4 (20.0)	2 (18.2)	8 (14.0)	0.446	6 (18.2)	2 (8.3)	8 (14.0)	0.291	6 (13.3)	0 (0)	1 (25)	1 (25)	0 (0)	0.832
	None	24 (92.3)	16 (80.0)	9 (81.8)	49 (86.0)		27 (81.8)	22 (91.7)	49 (86.0)		39 (86.7)	3 (100)	3 (75)	3 (75)	1 (100.0)	
Conversion	Yes	3 (11.5)	3 (15.0)	0 (0.0)	6 (10.7)	0.417	2 (6.1)	4 (17.4)	6 (10.7)	0.177	4 (8.9)	1 (33.3)	1 (25)	0 (4)	0 (0)	0.377
	None	22 (88.5)	17 (85.0)	11 (100.0)	50 (89.3)		31 (93.9)	19 (82.6)	50 (89.3)		41 (91.1)	2 (66.7)	3 (75)	4 (100)	0 (0)	
Abdominal drain	Used	5 (19.2)	10 (50.0)	3 (27.3)	18 (31.6)	0.079	7 (21.2)	11 (45.8)	18 (31.6)	0.048	10 (22.2)	2 (66.7)	3 (75)	2 (50)	1 (100.0)	0.044
	Not used	21 (80.8)	10 (50.0)	8 (72.7)	39 (68.4)		26 (78.8)	13 (54.2)	39 (68.4)		35 (77.8)	1 (33.3)	1 (25)	3 (50)	0 (0)	
Operative time (Mean & SD)	84.5±32.14	83.9±21.81	67.4±10.23	-	-	-	69.8 ±7.1	96.5 ±9.7	-	-	75.8 ±7.4	123.3 ±23.7	109.3 ±14.6	110.3 ±3.9	135	-
Drain Removal (Mean & SD)	2.5 ±1.096	2.5 ±1.42	1.3 ±0.53	2.3±0.9	-	-	1.1 ±0.5	3.1 ±1.3	-	-	2.1 ±1.5	3.5 ±0.7	3.3 ±2.2	2 ±0.7	-	-

There was a major difference seen in the number of days the patient stayed in the hospital post-operatively when they were managed differently by various approaches. The mean hospital stay post-early cholecystectomy was about two days, which increased significantly to 15 days with ERCP intervention. The mean postoperative stay post-interval cholecystectomy was about three days and with ERCP intervention of about two days. But, the total number of days the patient spent in the hospital had a mean of about 17 days for interval cholecystectomy, compared to 21 days for interval cholecystectomy with ERCP (Table 8).

**Table 8:** Hospital stay vs different plans of management EC - Early cholecystectomy, IC - Interval Cholecystectomy, ERCP - Endoscopic retrograde cholangiopancreatography

Management	Post op stay (Mean±SD)	Total stay (Mean±SD)
EC	1.93±2.08	5.88±4.37
EC with ERCP	15.33±15.94	21.66±18.44
IC	3.25±2.63	17.5±7.85
ERCP f/b IC	2.00±0.81	21.5±7.32
Cholecystostomy	7.00±0.00	9.00±0.00
Open Cholecystectomy	3.00±0.00	53.00±0.00

On the histopathological evaluation of the gallbladder, 86.1% were found to have features suggestive of chronic cholecystitis. Xanthogranulomatous cholecystitis was the second most common diagnosis on biopsy (8.8%). The most common type of stone found was mixed cholesterol type (61.4%), followed by brown (22.8%) and black (15.8%) (Table 9).

**Table 9:** Histopathological analysis

Histopathological analysis		No. of patients	Percentage
Histopathological Evaluation	Chronic Cholecystitis	49	86.1
	Xanthogranulomatous Cholecystitis	5	8.8
	Follicular Cholecystitis	1	1.8
	Hyperplastic Cholecystitis with intestinal metaplasia	1	1.8
	Necrosed gallbladder	1	1.8
Stone type	Black	9	15.8
	Brown	13	22.8
	Mixed cholesterol	35	61.4

There was no statistical difference found among the sub-groups of age based on the VAS score, presence of fever, SSI, recurrence of jaundice or any change in lifestyle of the patient on follow-up. But more than 70% of the patients who came for follow-up had an improved lifestyle (Table 10).

**Table 10:** Review on follow up of patients (Pearson Chi-Square Test) VAS - Visual Analogue Scale for pain

Review on follow-up		Age Groups			Total	p-Value
		60-69	70-79	≥80		
		n (%)	n (%)	n (%)	n (%)	
VAS (mean)		1.73	2.1	1.6	1.81	0.516
Fever	Present	2 (7.7)	1 (5.0)	0 (0.0)	3 (5.3)	0.631
	Absent	24 (92.3)	19 (95.0)	11 (100.0)	54 (94.7)	
SSI	Present	4 (15.4)	4 (20.0)	1 (9.1)	9 (15.8)	0.726
	Absent	22 (84.6)	16 (80.0)	10 (90.9)	48 (84.2)	
Jaundice	Present	1 (3.8)	1 (5.0)	0 (0.0)	2 (3.5)	0.763
	Absent	25 (96.2)	19 (95.0)	11 (100.0)	55 (96.5)	
Lifestyle	Decreased	5 (14.3)	9 (32.1)	2 (15.4)	16 (21.1)	0.367
	Improved	21 (60.0)	10 (35.7)	9 (69.2)	40 (52.6)	
	No change	0 (0.0)	1 (3.6)	0 (0.0)	1 (1.3)	
	Death	1 (2.9)	0 (0.0)	0 (0.0)	1 (1.3)	
	Lost of follow up	8 (22.9)	8 (28.6)	2 (15.4)	18 (23.7)	

## Discussion

One of the objectives of this study was to assess the natural history of gall stone disease in the senior citizen population. In this cohort, it was found that there was a higher incidence of GSD in females. Similar findings were found in other studies by Agrusa and Nielsen, where the incidence of GSD was found to be more in females than in males [9,10]. However, the difference in incidence was lesser as compared to that of this study. Contradictorily, Fukami found that the incidence of GSD was higher in males as compared to females [11]. Interestingly, the male population of this study was found to have more uncomplicated GSD whereas complicated GSD was found to be more common in the female group. On the contrary, in the study by Bailey, males had a higher incidence of complicated GSD. One of the possible reasons given by them for this could be that males have a lower tendency to attend hospitals [12].

Though no previous studies have been found comparing the prevalence of symptoms, the most common presenting complaint of the patients was pain abdomen (usually biliary colic). The least common complaint was yellowish discolouration of the skin. Jaundice is not common in the elderly because as age increases, the diameter of the common bile duct also increases which is consistent with our findings of the low incidence of jaundice (9.2%). This might have a role in the easy passage of gallstones through the duct. Due to this, symptoms associated with common bile duct stones were also lower. Similar results were found in the study by Kuang-Chun Hu [13].

In this study, it was found that patients with age greater than 80 years of age tended to have uncomplicated GSD when compared to the younger sub-groups, but this was not found to be statistically significant. This can again be attributed to the fact that as age increases, the diameter of the common bile duct also increases, and complications like choledocholithiasis, pancreatitis, and cholangitis usually take place due to the impaction of the stone in the duct. Due to this fact, there was a higher incidence of biliary pancreatitis in the younger group when compared to the elder group. Alternatively, in the study by Fukami, acute cholecystitis was more prevalent in the elderly population (>80 years) [11].

Even though no significant association was found among the different clinical presentations of GSD, there was a slightly positive trend toward the patient being pre-diabetic and diabetic. In previous studies like that done by Jun Lv, a 10-year prospective study, there was a strong correlation between diabetes and GSD incidence. Similar results were found in Sodhi et al. and Wang et al. All these studies were not able to confirm the direct casualty of GSD by the presence of diabetes, but because of a similar list of risk factors like obesity and insulin resistance, they were only able to conclude a correlation. Diabetes is known to decrease the mobility of the gallbladder because of diabetic neuropathy. There is an increased fasting volume of the gallbladder, compared to non-diabetic patients. Thus it contributes to bile stasis and gallstone formation. In our study, it was seen that prediabetic and diabetic states were associated with complicated GSD. Further research can be done in this regard [14-16].

More than 50% of the cases presenting with symptomatic GSD suffered from hypothyroidism. Our study showed a strong correlation between common bile duct stone and hypothyroidism. This association was also seen in studies by Laukkarinein and Song. It is suggested that T4 has pro-relaxation action on the sphincter of Oddi, and in hypothyroidism, there are increased chances of stagnation of bile in the common bile duct, thus leading to the formation of stones. Hypothyroidism is also associated with the formation of cholesterol stones. These two actions are known to increase the risk of stone formation [17,18].

To diagnose GSD, in addition to the history and clinical examination, radiological and blood investigations are required. The most common complaint of the patient was pain abdomen associated with dyspepsia and vomiting. About 40% of the total cases presented to the hospital with an acute history, out of which only 13 patients were diagnosed with acute cholecystitis, 10 had biliary pancreatitis, and two had cholangitis. In a study by Magnuson, similar results were found when comparing the elderly population to the younger one, though the incidence of acute calculus cholecystitis was lower in our study [19].

Out of 76 patients, 75 had undergone abdominal ultrasonography and 74 patients were found to have either one or multiple gallstones in the gallbladder. In the one patient where no gallstones were found on ultrasound, a diagnosis of biliary pancreatitis was made and he underwent Contrast Enhanced Computerised Tomography (CECT) scan of the abdomen based on their history, clinical findings, laboratory reports, and presence of a dilated CBD on ultrasound. This patient underwent ERCP stenting and retrieval of stones/sludge. 11 patients also underwent MRCP when the anatomy of the biliary system needed further evaluation. Grossly, the findings of the ultrasound and MRCP were somewhat similar, though there was an underreporting of about 30% regarding the diameter of the CBD. This further weakens the use of MRCP in the case of GSDs, where ultrasound can provide major information required to diagnose the disease [20,21].

Blood samples were also sent to augment the differential diagnosis and as part of the pre-operative evaluation. A quarter of these patients were found to have increased SGPT or SGOT levels. Among these, 90% were found to be marginally higher than the normal range and did not change our management plan. A total of 10% of the patients had hyperbilirubinemia, whereas 22% were diagnosed with a stone in the common bile duct, which indicates the significance of these tests as more of screening value or prognosticative value other than for diagnosis.

**Table 11:** Reference values for lab investigations

Test	Reference values
Hemoglobin	13.5-17.5 gm/dL (male); 11.5-15.5 gm/dL (female)
Total Leucocyte count	4-11 x 10^9 ^cells/L
SGOT	<50 IU/L (male), <35 IU/L (female)
SGPT	<50 IU/L (male), <35 IU/L (female)
Bilirubin	0.3-1.2 mg/dL
Direct bilirubin	<0.2 mg/dL
Indirect bilirubin	<0.5 mg/dL
ALP	30-120 IU/L
HbA1c	4-6.2%
TSH	0.3-3.6 mIU/L
FT3	2.2-4.2 pg/mL
FT4	0.8-1.7 ng/dL
Amylase	28-100 IU/L
Lipase	<67 IU/L

Once the diagnosis of either complicated or uncomplicated GSD was made, the appropriate management plan was made for each patient based on their clinical status and after discussing the plan with the patient and their family members. Out of 76 patients, only 57 patients underwent surgical intervention. Out of the remaining 19 patients who did not, 18 were optimised in their primary admission and were planned for interval cholecystectomy but lost to follow-up because of the ongoing COVID-19 pandemic of 2020. Out of these 19 patients, one had surgery deferred due to uncontrolled diabetes and was lost to follow-up later on.

In the 57 patients who were operated on, there was no significant difference seen in the intraoperative findings of the patients in different age groups. Because there was no difference in difficulty level based on the operative time, we can concur that the patient's age is not a risk factor for a “difficult” surgery. Similar results have been noted in studies by Loureiro and Trust where they concluded that in a hemodynamically stable patient with features suggestive of mild acute cholecystitis, or even mild biliary pancreatitis, age has no effect on the outcomes of the laparoscopic cholecystectomy and should be considered as the primary modality when treating the senior citizens [22,23].

In a study by Nielsen et al, the average conversion rate for laparoscopic cholecystectomy to open cholecystectomy for patients aged more than 65 years of age was about 15.9%, which was higher than our result of 10.5%. The most common reason for conversion found in our study was adhesions, whereas in their study it was age more than 80 years of age, acute cholecystitis and previous abdominal surgeries. Even though Nielsen concluded that age is a risk factor for conversion, there was no significant difference in the different sub-groups in our study. This might be because most patients of ages >80 years had presented with uncomplicated GSD [10].

Interestingly, this study found that patients older than 80 years of age had fewer cases of adhesions, contracted gallbladder, and even had a lower incidence of complications and a lower conversion rate. This was probably because this group of patients was suffering from a milder form of GSD. Because of this, the mean operative time for this age group was also lower. Though this was not statistically significant.

As expected, there was a higher incidence of adhesions in complicated GSD due to active and or chronic inflammation associated with the condition which led to higher chances of gallbladder being contracted. Even though there was a 14% incidence of intra-operative complications, iatrogenic perforation of the gallbladder during dissection was the most common one; none of these iatrogenic perforations led to conversion to open surgery. The most common reason for conversion was to prevent iatrogenic injury due to dense adhesions in the operative site. There was a trend toward placing a drain in the abdomen, and the most common reasons were to check for any bile leak from the distal cystic duct stump and drain out any peritoneal contamination.

In our study, it was found that patients who underwent interval cholecystectomy had a 100% incidence of the presence of adhesions, this can be attributed to the fact that these patients were initially suffering from complicated GSD, and because of increased localized inflammation, dense adhesions were found in the surgery. Moreover, for this reason, there were increased chances of complications, conversion rate, and increased use of drains in the surgery. A study by Serna concluded that there is no difference in early cholecystectomy and interval cholecystectomy outcomes in cases of mild to moderate acute cholecystitis [24]. A study by Fuks went on further to conclude that the outcomes of early cholecystectomy in patients less than the age of 75 years had a similar result compared to patients more than 75 years of age [25]. However, Nikfarjam had asserted that elder patients (>80 years) had a worse postoperative prognosis when compared to their younger counterparts and hence should be managed optimally before taking to the operating theatre in acute settings [26]. In our study, there was a trend towards lesser operative time in early cholecystectomy for acute cholecystitis as compared to interval cholecystectomy, but further research is required to validate this observation. On histopathological evaluation, similar findings were noted compared to studies like that by Khan. Interestingly, in this study, the incidence of xanthogranulomatous cholecystitis was less than half of what was found in our study. The worldwide incidence of xanthogranulomatous cholecystitis is about 1%-3%, whereas, in India, it is about 8.8%, which matched our data. There have been several theories as to why India has such a high number of cases of xanthogranulomatous cholecystitis; the most accepted one is the high number of cases of GSD in India. Dedicated research can find the true root cause of these findings [27,28].

A study by Cotta conclude that the most common variety of gallstones is of cholesterol type (mixed and pure) (60%), followed by composite (21%), black-pigmented (8.5%) and brown pigmented (6.5%) stones. Though the incidence of mixed cholesterol stones in our study was found similar to this, the number of pigmented gallstones was more than double for black and triple for brown [29].

Limitations

One of the major limitations of this study was its small sample size. Due to the presence of the COVID-19 pandemic during the period of study, a number of patients were lost to follow-up. The average number of days of hospital stay was increased, as some patients were discharged due to COVID-19 infection and readmitted after the quarantine period. The period to obtain the COVID-19 reverse transcription polymerase chain reaction (RTPCR) report in our institute was around two to three days, and it was institute protocol to get this done at the time of admission and just before surgery. If the patient turned out to be COVID-19 positive, then the patient was discharged for a quarantine period of two weeks and advised for readmission during which a repeat COVID-19 RTPCR test was carried out. This exercise in turn increased the total period of days of hospitalization.

## Conclusions

At the end of the study, it was found that GSD was is more commonly seen even in females of more than 60 years of age than males, though uncomplicated GSD was more common in men. There was a trend seen towards uncomplicated as the age increases though it was statistically insignificant. GSD is diagnosed similarly to the younger population with USG and lab investigations although some might require MRCP in cases of doubts of malignancy. In cases of complicated GSD, there were higher chances of a longer operative period, difficult surgery, and longer post-operative hospital stay, and some patients also required additional procedures like ERCP thus increasing hospital stay. There was a trend seen towards complicated GSD being more common with diabetes mellitus and hypothyroidism but it was not statistically significant. There was no relationship found between the presence of hypertension and GSD.

Earlier surgical intervention in form of laparoscopic cholecystectomy can be beneficial to the patient if diagnosed with gallstones. Patients of this age group may not be over investigated if a benign pathology is suspected. In the case of mild acute cholecystitis and mild biliary pancreatitis, early cholecystectomy can be the intervention of choice and age should not be a limiting factor for the surgery. Xanthogranulomatous cholecystitis has been found at a higher prevalence rate in this study and other studies, but the true pathogenesis of this entity is not known. Further research can be done in this regard. This might be one of a kind study in the Indian subcontinent, much more detailed research can be done to further remove the fear of bad outcomes in “old age” from the surgeon and the anaesthesiologist.

## References

[1] Lammert F, Gurusamy K, Ko CW (2016). Gallstones. Nat Rev Dis Primers.

[2] Hendrickson M, Naparst TR (2003). Abdominal surgical emergencies in the elderly. Emerg Med Clin North Am.

[3] Heger E, Lammert F (2014). Biliary diseases in the elderly (Article in German). Z Gastroenterol.

[4] Bergman S, Al-Bader M, Sourial N (2015). Recurrence of biliary disease following non-operative management in elderly patients. Surg Endosc.

[5] Reshetnyak VI (2012). Concept of the pathogenesis and treatment of cholelithiasis. World J Hepatol.

[6] Yokota Y, Tomimaru Y, Noguchi K (2019). Surgical outcomes of laparoscopic cholecystectomy for acute cholecystitis in elderly patients. Asian J Endosc Surg.

[7] Hegazy TO, Soliman SS (2018). Early versus interval laparoscopic cholecystectomy for treatment of noncomplicated acute calcular cholecystitis. Egypt J Surgery.

[8] Parmar AD, Sheffield KM, Adhikari D (2015). Preop-gallstones: A prognostic nomogram for the management of symptomatic cholelithiasis in older patients. Ann Surg.

[9] Agrusa A, Romano G, Frazzetta G, Chianetta D, Sorce V, Di Buono G, Gulotta G (2014). Role and outcomes of laparoscopic cholecystectomy in the elderly. Int J Surg.

[10] Nielsen LB, Harboe KM, Bardram L (2014). Cholecystectomy for the elderly: no hesitation for otherwise healthy patients. Surg Endosc.

[11] Fukami Y, Kurumiya Y, Mizuno K, Sekoguchi E, Kobayashi S (2014). Cholecystectomy in octogenarians: be careful. Updates Surg.

[12] Bailey KS, Marsh W, Daughtery L, Hobbs G, Borgstrom D (2022). Sex disparities in the presentation of gallbladder disease. Am Surg.

[13] Hu KC, Chu CH, Wang HY (2016). How does aging affect presentation and management of biliary stones?. J Am Geriatr Soc.

[14] Lv J, Yu C, Guo Y (2017). Gallstone disease and the risk of type 2 diabetes. Sci Rep.

[15] Sodhi JS, Zargar SA, Khateeb S (2014). Prevalence of gallstone disease in patients with type 2 diabetes and the risk factors in North Indian population: a case control study. Indian J Gastroenterol.

[16] Wang F, Wang J, Li Y (2019). Gallstone disease and type 2 diabetes risk: a Mendelian randomization study. Hepatology.

[17] Laukkarinen J, Kiudelis G, Lempinen M (2007). Increased prevalence of subclinical hypothyroidism in common bile duct stone patients. J Clin Endocrinol Metab.

[18] Song ST, Shi J, Wang XH (2020). Prevalence and risk factors for gallstone disease: a population-based cross-sectional study. J Dig Dis.

[19] Magnuson TH, Ratner LE, Zenilman ME, Bender JS (1997). Laparoscopic cholecystectomy: applicability in the geriatric population. Am Surg.

[20] Bahram M, Gaballa G (2010). The value of pre-operative magnetic resonance cholangiopancreatography (MRCP) in management of patients with gall stones. Int J Surg.

[21] (2022). Does preoperative magnetic resonant cholangiopancreatography (MRCP), improve the safety of laparoscopic cholecystectomy?. https://www.sciencedirect.com/science/article/pii/S2405857217300906.

[22] Loureiro ER, Klein SC, Pavan CC, Almeida LD, da Silva FH, Paulo DN (2011). Laparoscopic cholecystectomy in 960 elderly patients. Rev Col Bras Cir.

[23] Trust MD, Sheffield KM, Boyd CA, Benarroch-Gampel J, Zhang D, Townsend CM Jr, Riall TS (2011). Gallstone pancreatitis in older patients: are we operating enough?. Surgery.

[24] De la Serna S, Ruano A, Pérez-Jiménez A (2019). Safety and feasibility of cholecystectomy in octogenarians. Analysis of a single center series of 316 patients. HPB (Oxford).

[25] Fuks D, Duhaut P, Mauvais F (2015). A retrospective comparison of older and younger adults undergoing early laparoscopic cholecystectomy for mild to moderate calculous cholecystitis. J Am Geriatr Soc.

[26] Nikfarjam M, Yeo D, Perini M, Fink MA, Muralidharan V, Starkey G (2014). Outcomes of cholecystectomy for treatment of acute cholecystitis in octogenarians. ANZ J Surg.

[27] Khan S, Jetley S, Husain M (20131). Spectrum of histopathological lesions in cholecystectomy specimens: a study of 360 cases at a teaching hospital in South Delhi. Arch Int Surg.

[28] Hale MD, Roberts KJ, Hodson J, Scott N, Sheridan M, Toogood GJ (2014). Xanthogranulomatous cholecystitis: a European and global perspective. HPB (Oxford).

[29] Cotta F (2008). Classification, composition and structure of gallstones. Relevance of these parameters for clinical presentation and treatment. Biliary Lithiasis: Basic Science, Current Diagnosis and Management.

